# Drinking water quality in Indigenous communities in Canada and health outcomes: a scoping review

**DOI:** 10.3402/ijch.v75.32336

**Published:** 2016-07-29

**Authors:** Lori E. A. Bradford, Lalita A. Bharadwaj, Udoka Okpalauwaekwe, Cheryl L. Waldner

**Affiliations:** 1 School of Public Health, University of Saskatchewan, Saskatoon, SK, Canada; 2 Department of Large Animal Clinical Sciences, Western College of Veterinary Medicine, University of Saskatchewan, Saskatoon, SK, Canada

**Keywords:** drinking water quality, Indigenous communities, health, Canada, First Nations, scoping review

## Abstract

**Background:**

Many Indigenous communities in Canada live with high-risk drinking water systems and drinking water advisories and experience health status and water quality below that of the general population. A scoping review of research examining drinking water quality and its relationship to Indigenous health was conducted.

**Objective:**

The study was undertaken to identify the extent of the literature, summarize current reports and identify research needs.

**Design:**

A scoping review was designed to identify peer-reviewed literature that examined challenges related to drinking water and health in Indigenous communities in Canada. Key search terms were developed and mapped on five bibliographic databases (MEDLINE/PubMED, Web of Knowledge, SciVerse Scopus, Taylor and Francis online journal and Google Scholar). Online searches for grey literature using relevant government websites were completed.

**Results:**

Sixteen articles (of 518; 156 bibliographic search engines, 362 grey literature) met criteria for inclusion (contained keywords; publication year 2000–2015; peer-reviewed and from Canada). Studies were quantitative (8), qualitative (5) or mixed (3) and included case, cohort, cross-sectional and participatory designs. In most articles, no definition of “health” was given (14/16), and the primary health issue described was gastrointestinal illness (12/16). Challenges to the study of health and well-being with respect to drinking water in Indigenous communities included irregular funding, remote locations, ethical approval processes, small sample sizes and missing data.

**Conclusions:**

Research on drinking water and health outcomes in Indigenous communities in Canada is limited and occurs on an opportunistic basis. There is a need for more research funding, and inquiry to inform policy decisions for improvements of water quality and health-related outcomes in Indigenous communities. A coordinated network looking at First Nations water and health outcomes, a database to store and create access to research findings, increased funding and time frames for funding, and more decolonizing and community-based participatory research aimed at understanding the relationship between drinking water quality and health outcomes in First Nations communities in Canada are needed.

Although Canada is recognized around the world for its natural wealth of fresh water (1), many Indigenous communities experience challenges to accessing safe drinking water. Literature suggests the water crisis in Indigenous communities is reflective of a host of unresolved matters that speak to issues of colonization, inequity, justice and institutional trends within governing and funding bodies in Canada (2).

Provincial water regulations do not apply to Indigenous communities. A complex tri-departmental federal structure consisting of Aboriginal Affairs and Northern Developmental Canada, Health Canada and Environmental Canada shares responsibility for safe delivery of drinking water. First Nations community leadership groups such as Chief and Councils must assume 20% of the costs for water infrastructure, and operations and maintenance, and are additionally tasked with monitoring water safety and ensuring the presence of trained operators. This complex governance structure has led to gaps in drinking water regulation in Indigenous communities across Canada. The Federal Government passed Bill S-8, the Safe Drinking Water for First Nations Act into law in 2013 as a means to address the regulatory gaps. It is unclear whether this Bill will significantly mitigate regulatory issues or lead to improvements in drinking water quality for Indigenous communities (3).

To Indigenous people, water is more than a commodity or a necessity for physical survival. In some Indigenous worldviews, water is considered a gift from the Creator, the lifeblood of Mother Earth and a spiritual resource that must be respected and kept clean (2,4–6). Government reports and assessments, as well as case-study reviews by non-profit organizations, highlight and identify imbalances in the provision of safe drinking water between Indigenous and non-Indigenous communities (7–9). Waterborne infections are more common in Indigenous communities compared to the national average, and 30% of Indigenous community water systems are described as “high risk.” Inequities in the provision and access to reliable and sustainable sources of drinking water leave Indigenous communities vulnerable to waterborne diseases, potential exposures to chemical contaminants and associated health effects. As of November, 2015, there were 138 Drinking Water Advisories (DWA) in effect in 94 First Nations communities across Canada, excluding British Columbia (1,10).

In 2005, Assembly of First Nations National Chief Phil Fontaine indicated that a first step in ensuring the health of Indigenous people and their communities was to address critical and urgent priorities such as safe drinking water on reserves. This scoping review on the nature and extent of academic and non-academic research on the topic of drinking water quality and Indigenous health was prompted by current reports on Indigenous health and the emergence of regulatory water policy. The results summarize documented challenges in access to safe drinking water for Indigenous communities in Canada, associated health issues (physical, psychological and social), methodological challenges and existing gaps in the literature (11). It is recognized that water security is an issue of both quality and quantity (12). This work focuses on aspects of water quality; however, there is an emerging body of research illustrating the effects of inadequate water quantity and resulting health outcomes in Indigenous communities in the north (13,14).

## Investigation

### Methods

The scoping review framework outlined by Arksey and O'Malley (15) and advancements by Pham et al. (16) were used. The steps included identification of the research question; identification of relevant articles and article selection; charting of the data; collating, summarizing and reporting of the results; and conducting a consultation exercise among the co-authors of this manuscript. The review process was summarized as sequential steps; however, the review process was not linear, some steps were repeated to ensure a comprehensive assessment of the literature. A scoping review was selected over a systematic review because the purpose was not to extract data, or formally assess the quality of studies and make specific conclusions, rather, the review sought to identify challenges faced by researchers and gaps in the literature (17).

### The research question

A research team including a graduate student in public health, postdoctoral social scientist, toxicologist and epidemiologist was established. The team formulated the research questions, the overall study protocol and selection criteria for this review. The scoping review was guided by the questions: How do challenges (i.e. historical, social, political, cultural and environmental) associated with drinking water impact the health of Indigenous communities? And what gaps in the research are evident?

### Data sources and search strategy

A comprehensive search strategy was designed with the assistance of a university librarian. Key search terms were developed and mapped with online databases prior to the article search. The initial search was carried out from 9 to 25 February 2015. Electronic databases that covered a wide range of disciplines were used initially, which include MEDLINE/PubMED (biomedical sciences, 2000 – present), Web of Science (multidisciplinary, 2000 – present), SciVerse Scopus (multidisciplinary 2000 – present) and Google Scholar. Search queries consisted of the keywords: Indigenous communities (and synonyms), drinking water, health and challenges tailored to the requirements of each database (see Table I).

**Table I:** Keywords (with synonyms) and syntax used for literature search

**#1**	**Drinking water** Drinking water quality OR water quality OR potable water OR healthy water OR drinkable water OR drink water OR drink OR safe water OR water OR suitable water OR palatable water OR edible water OR tap water OR fresh water OR water supply
**#2**	**Indigenous communities** Indigenous people OR Indigenous OR Aborigine OR Aboriginal OR Indigenous OR Native(s) OR Indigen* OR Indigenous people OR First Nations OR Metis OR Inuit Or Inuk
**#3**	**Health** Health outcomes OR wellness OR well being OR physical health OR mental health OR social health
**#4**	**Challenges** Challenge* OR limitation* OR gap* OR barrier* OR obstacle*
**#5**	**Canada** Canada OR North America
**#6**	#1 AND #2 AND #3 AND #5
**#7**	#6 AND #4

A Google web search was also conducted using the search strings First Nations AND Drinking water AND Health AND Canada to identify grey literature. A decision to screen the first 100 hits from the Google search was made a priori, considering the time required to screen each article. This theory is based on evidence that further screening is unlikely to yield many more relevant articles. Additional websites were searched manually (Table II).

**Table II:** Additional websites used to identify grey literature for the scoping review

Source	URL/link
Public Health Grey Literature Sources (via OPHLA)	(www.ophla.ca/pdf/Public Health Grey Literature Sources.pdf)
Canadian Electronic Library: Canadian Public Policy Collection (CPPC)	www.library.usask.ca/find/connect.php?code=CELCPPC
Healthcare Standards Directory Online	www.library.usask.ca/find/connect.php?code=HCS
Centre for Indigenous Environmental Resources	www.yourceir.org
University repository for literature in Indigenous studies	Iportal.usask.ca

**Fig. 1:**
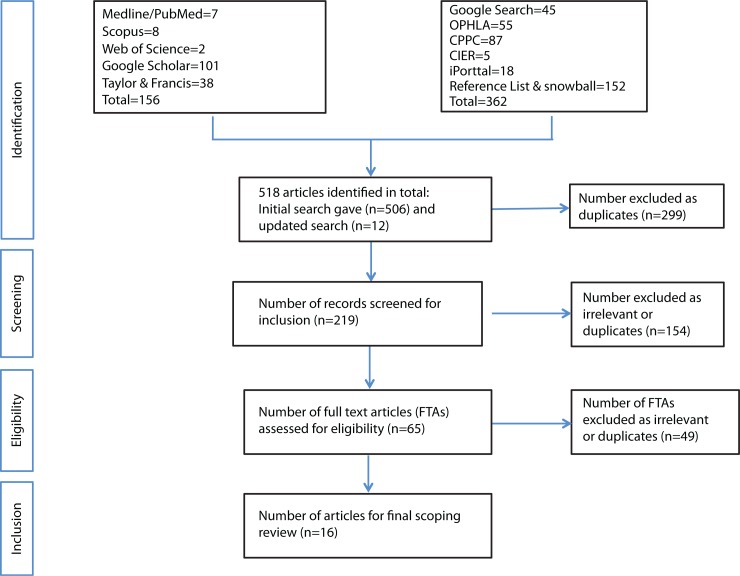
PRISMA (21) flowchart of study selection process. OPHLA: Ontario Public Health Libraries Association. CPPC: Canadian Public Policy Collection. CIER: Center for Indigenous and Environmental Resources (see Table II).

On 25 February 2015, an initial list of articles that met eligibility criteria was created from all articles identified during the initial scope (Fig. 1). Subsequently, articles and their citations were manually searched to identify any additional articles for inclusion in the scoping review. Citations within articles were searched if they appeared relevant to the scoping review. This snowball technique was adopted to ensure a comprehensive and thorough search. Another search was carried out on 15 March 2015 using publisher's online search bars, in addition to the four bibliographic databases and other literature sources listed above. Eight months later, a second search was carried out again to ensure exhaustiveness. These searches applied the same search strings, keywords and date restrictions as shown in Table I.

All citations, along with abstracts, were imported or manually entered into reference managers Endnote X7 (18) and Mendeley 13.8 (19). Associated full-text articles (FTAs) were thereafter added. Duplicates were removed manually after assembling citations.

### Eligibility criteria and study selection

The scoping selection was limited to peer-reviewed documents from Canada subject to three inclusion criteria. The first criterion for inclusion involved keywords; peer-reviewed journal articles, theses, government and technical reports with the keywords (and synonyms as listed in Table I) and combinations of these terms were selected. The second criterion was the timeframe of publication. Only papers published between the year 2000 and 2015 were selected. Finally, only English and English/French documents were selected for inclusion.

A two-stage process was used to assess the relevance of articles identified from the search. After the initial article collation and deduplication, articles were manually screened by checking their titles and abstracts for identified keywords. Thereafter, the FTAs with two or more keyword combinations were retrieved for further screening. The second stage involved reading all FTAs retrieved from the initial screening to identify articles that discussed issues related to drinking water quality in Indigenous communities, First Nations in Canada, health outcomes and other challenges to safe drinking water among Indigenous communities.

### Data charting and summary

A Microsoft Access database (20) was used for data entry validation and coding. Data extracted from the selected articles included author(s), year of publication, title, design, type, location and type of Indigenous community. Other information extracted from the selected articles included summary of the findings, reported health outcomes, drinking water assessment and quality, associations and comparisons, recommendations and limitations. Articles were labelled by letter. The results below include proportions of articles with similar findings, as well as individually identified articles for reference (i.e. 12/16 articles were published in academic peer-reviewed journals; these included articles A–C, E, G, H and K–O).

### Consultation exercises

At several intervals during the search and data inclusion phases, consultation exercises were conducted with the research team. Input on keyword selection, inclusion criteria and relevance of selections for each search strategy was provided during face-to-face and Email communications. Clarifications on methodological approach and tools were sought by the graduate student. The postdoctoral researcher conducted the second search, analysed data at the thematic level and provided direction on article summaries and tables.

## Results

### Overview of selected studies

A total of 518 articles were retrieved from the overall search; 156 from the bibliographic search engines and 362 from grey literature. Following deduplication and relevance screening, 65 articles were found to meet the three first-level eligibility criteria (based on title and abstract). All 65 FTAs were reviewed for inclusion based on the second-level eligibility criteria (relevance to research question). Of the 65 FTAs, 49 articles did not meet the second-level eligibility criteria leaving a total of 16 articles for inclusion into the final scoping review.

The results section has three subsections beginning with the characteristics of the studies in the sample. Secondly, we report on methodological strengths and limitations of both the studies themselves and the sample as a whole. Thirdly, we explore the specific content of the studies, overall themes and gaps in research examining health outcomes and water in Indigenous communities.

### Study characteristics

#### Descriptive summaries of study characteristics

The general characteristics for the articles from the search are shown in Tables III and IV. Most articles were published in academic journals (12/16), after the year 2010 (11/16) and had a mean study duration of 30 months. Two theses (F and P) and two government reports (I and J) also met the inclusion criteria. The documents ranged from 4 (N) to 129 pages (I).

**Table III:** Summary of articles included in scoping review (n=16)

*ID* Authors Year (Citation #)	Topic	Design: method Data type *N* *Response rate*	Site^a^ (FN=First Nation)	Summary of findings	Limitations
*A* Tam et al. 2015 (22)	Iodine status of Eeyou Istchee community members of northern Quebec, Canada, and potential sources	Quantitative: Cohort study Primary data *750 participants* *No data on what % of those selected for the study declined*	Six Cree FNs in PQ	Correlation between higher consumption of tap water (in First Nation communities) and local spring water (in the bush) and lower levels of Urinary Iodine Concentration (UIC) and increased risk of iodine deficiency disorders -suggesting these water sources contained lower iodine levels.	Individual variations in UIC measurement. Iodine content in water sources was not analysed with reference to iodine content across other Canadian water sources.
*B* Dupont et al. 2014 (23)	Drinking water management: Health risk perceptions and choices in FNs and non-FNs communities in Canada	Quantitative: Participatory research Primary data *301 FN (ON)* *86 FN (SK)* *1,307 Non-FN* *23.5% for non-FN* *41% for SK FN community. Response rates not reported for ON FN communities*.	Four FNs communities: Six Nations; New Credit; Oneida FN ON Muskoday FN SK	Explored the perspectives on health risks from tap water and bottle water. ON FN more likely to believe bottled water safer than tap water, more likely to express water and health concerns related to tap water consumption and to report illnesses related to drinking tap water, and more likely to spend more on bottled water. Residents of the Saskatchewan FN community were less likely to than non-FN respondents for all parameters above.	Selection bias or volunteer bias for survey responders. Could not evaluate the response rate for Oneida, ON surveys.
*C* Hanrahan et al. 2014 (24)	Water insecurity in Indigenous Canada: A case study of Illness, Neglect, and Urgency	Mixed methods: Case study Primary data Water testing: one PDWU and seven dug out wells Interviews/Focus Groups: *One community Number of participants not reported*	Black Tickle-Domino NL	Water samples contain high level of carcinogenic disinfectant by-products. Turbidity was high (no figures given), which may have been due to its high iron content and natural organic matter. Black Tickle residents confirmed high rates of diabetes, obesity (80% of female study participants) owing to high rates of sugary beverage consumption as alternative to drinking water.	Not peer-reviewed. No limitations reported. Conference proceeding. Methods not documented in detail.
*D* McClymont Peace and Myers 2012 (25)	Community-based Participatory Process, Climate Change and Health Adaptation Program for Northern First Nations and Inuit in Canada	Qualitative: Participatory research Evaluation study *15 visits to three communities (five visits in each) and three capacity building workshops after the visits*	Whitehorse, YK; Yellowknife, NT, and Ottawa, ON; with participation from FNs, government representatives, and other related organizations across YK, NT, ON	Evaluation of programs and capacity building workshops for northerners to promote research tools and policy measures on climate change and water quality among northern Indigenous communities.	Funding and delays in execution of the workshops. No specific data collected or reported on water and health.
*E* Spence and Walters 2012 (26)	Risk perception and drinking water in a vulnerable population	Quantitative: Cross-sectional Survey Secondary data from 2001 Aboriginal Peoples Survey *17,506 individuals from 123 FN reserves in Canada*	FNs across Canada selected for the 2001 Aboriginal Peoples Survey	Using multiple logistic regression models, assessed the determinants of risk perception of drinking water in the home among FN on reserve in Canada. Variables associated with greater perception of risk for drinking water in the home included being female, being highly educated, having children less than 15 years of age, being in poor health, having less attachment to Aboriginal culture, living in a residence requiring major repairs, reporting water contamination during the previous year or being uncertain of the contamination status of water, and residing in specific geographic areas.	Smaller reserves were not included. Limitations in survey tool on concepts and measures associated with risk perception of drinking water. Under-reporting of FN perception regarding health risk related to drinking water based on survey structure.
*F* Harbinson 2012 (8)	Graduate level Independent Study project: An Analysis of Water Quality and Human Health Issues in First Nations Communities in Canada	Qualitative: Case study Content analysis Secondary compilation of current literature from local agencies, government and other documents *No data analysis*	Case studies from three First Nations communities summarized: Kashechewan ON Yellow Quill SK Fort Chipewyan AB	Results showed higher rates of certain diseases among First Nation communities than other Canadian citizens, reported to be related to exposure to poorer water quality.	Not a peer-reviewed. Where information was missing or authors deemed it to have questionable authenticity, this was noted in the report. The authors recommended caution when generalizing findings of the case studies to other Canadian populations or
					First Nations communities, as the factors influencing the water and health quality could be significantly different.
*G* Harper et al. 2011 (27)	Weather, Water Quality and Infectious Gastrointestinal Illness in Two Inuit Communities in Nunatsiavut, Canada: Potential. Implications for Climate Change	Quantitative: Participatory research Primary and Secondary data *Ecological, water quality and health records data*. *No individual person or household data collected*.	Two Inuit communities: Nunatsiavut: Nain and Rigolet in NL	Study analysed and compared data on weather, water quality and health in Nunatsiavut FN community. Poisson regression was used to examine associations between weather events and infectious gastrointestinal disease (IGI) clinic visits. Results showed a higher number of IGI related clinic visits in the summer and fall months) and when high levels of water volume input 2 and 4 weeks prior.	Missing weather and water quality data. Inability to identify the origin of gastro illness. Gender differences in illness were difficult to sort out from clinic use. Not possible to exclude patients who visited more than once for same illness.
*H* Patrick 2011 (9)	Uneven access to safe drinking water for First Nations in Canada: Connecting health and place through source water protection	Theoretical/Qualitative: Case and Content analysis *No primary or secondary data analysis*	Neskantanga ON	Exploration of health promotion through examination of access to safe drinking water showed that source water protection in addition to water quality monitoring through technology is vital in maintaining health of First Nation communities.	No limitations reported.
*I* Ekos Research Associates 2011 (28)	Technical report (Health Canada): Perceptions of Drinking Water Quality in FN Communities and General Population	Quantitative: Cross-sectional Primary data *674 + 227 FN residents on reserves and 706 residents from non-FN small communities* *15% response rate for small communities and 21% response rate for FN reserves. Missing data not reported*.	FNs and other small communities across Canada including all provincial regions and the territories	Underlines the difference in confidence levels between FN and non-FN for drinking water quality. Analysis showed FN residents are less confident (variable figures with reference to water source, water quality and households) about the quality of water they received than residents from non-FN communities.	Not peer-reviewed. Brief telephone interviews used to collect data. Language barriers.
*J* Anderson 2010 (4)	Technical report: Aboriginal Women, Water and Health: Reflections from 11 First Nations, Inuit and Métis grandmothers	Qualitative: Participatory research Interviews Primary data *11 grandmothers*	BC, AB, SK, ON, Nunavut, Labrador.	Interviews highlighted the importance of water as a spirit and its traditional role in promoting health.	Not peer-reviewed No limitations reported except for use of phone interviews rather than in person for women from Labrador.
*K* Lebel and Reed 2010 (29)	Case study of the Capacity of Montreal Lake, Saskatchewan to Provide Safe Drinking Water by Applying a Framework for Analysis	Mixed methods: Case study, interviews, public workshop, document analysis, water quality testing *1 community* *5 interviewed and 16 in workshop*	Montreal Lake FN, SK	Established an analytical framework for evaluating the capacity of FNs community to provide safe drinking water, and sustaining its water quality; applied framework to case study community where no serious deficiencies in the management of drinking water were found.	Applying the framework (built from indicators from literature in FN and non-FN communities) in a community where drinking water was a risk may have resulted in bias.
*L* Bernier et al. 2009 (30)	On-site microbiological quality monitoring of raw source water in Cree community of Mistissini	Quantitative: Case study Primary water quality data collected on sites *74 water samples from 12 water sources plus 21 samples from portable water containers* *24 households*	Cree people of Mistissini PQ	Assessed the use of multiple indicators (*E. coli* and *Enterococci*) in microbiological water quality monitoring of source water in a Cree community. Results revealed that several storage practices decreased the microbiological quality of raw source water and thus drinking water-related health outcomes.	Technological constraints limiting the use of culture-based methods in water quality monitoring.
*M* Simpson et al. 2009 (6)	The Responsibilities of Women: Confronting Environmental Contamination in traditional territories	Decolonizing Qualitative: Participatory research Primary data from culturally-sensitive and community-owned focus groups *Number of participants not reported*	Two FN communities: Asubpeechoseewagong Netum Anishinabek (Grassy Narrows) and Wabauskang, ON	Described experiences and perspectives of women elders on water and land contamination and impact to health of Indigenous communities. No causal relationship was reported however scenarios of environmental contamination and health outcomes were highlighted.	No limitations reported.
*N* Smith et al. 2007 (31)	Public Health Evaluation of drinking water systems for First Nations reserves in Alberta, Canada	Quantitative: Cross-sectional Primary data from water samples, and survey of risk evaluations. *56 water systems and Environmental Health Officer and water treatment plant operator for each community*	FNs across AB	Risk perception evaluation of 56 water supply systems showed 50 to be termed as high risk, five medium risk and one low risk using numerical score systems and health risk indicators.	Took conservative approach to situations of cumulative risk, and underreporting of gastrointestinal illness, and likely protective immunity from long-term exposure were limitations.
*O* Jin and Martin 2003 (32)	Hepatitis A Among Residents of First Nations Reserves in British Columbia, 1991–1996	Quantitative: Cohort Secondary data from health agency records *50,787 records in the 1994 water and sewages survey and 49,756 records from the 1993 survey*	257 FN reserves belonging to 197 bands across BC	Incidence of hepatitis A among residents of FN reserves twice as high as the crude incidence in the general population of BC for study period An association between increase hepatitis A incidence and greater numbers of persons per housing unit (i.e. crowding and water use) showed a relative risk of 6.7 (95% CI 4.3–10.5).	Small sample
*P* Maclean 2002 (33)	Thesis: Source Water Characteristics and the Incidence of Gastroenteritis in Aboriginal Communities	Mixed method: Cohort Primary data from surveys *74 + 32 +37+28 = 171 reflecting an average retention rate of 78.5% at the end of the study*	Bonaparte Band, Neskonlith Band, and Kamloops Band BC	Explored drinking water sources and unreported diarrheal illness and the potential relationships between water treatment types (chlorination and filtration) and the incidence of unreported diarrheal illnesses, in the three First Nation communities. Incidence rates of unreported diarrhoeal illnesses from cluster groups were comparatively low in residents with disinfection only, shallow infiltration and drilled wells with no significant difference in incidence between Kamloops and Neskonlith communities (0.84 per person year and 0.80 per person year, respectively).	Limited length of study. Uncontrolled use of multiple water sources offers protection. Under-reporting of symptoms. Selection bias minimized by random sample. Non-blinding of study subjects. Failure to pre-screen study subjects for pre-existing health conditions and immune deficiencies may have also biased results.

a Provincial and territorial abbreviations used: AB: Alberta, BC: British Columbia, NL: Newfoundland and Labrador, NT: Northwest Territories, ON: Ontario, PQ: Quebec, SK+ Saskatchewan, YK: Yukon Territory.

**Table IV:** General attributes of publications included in the scoping review (n=16)

Characteristic	Number (n=16)	%	Article ID numbers^a^
Publication year			
2000–2004	2	13	(O, P)
2005–2009	3	19	(L, M, N)
2010–June 2014	11	69	(A–K)
Publication type			
Journal article or conference proceeding	12	75	(A–E, G, H, K–O)
Thesis or academic report	2	13	(F, P)
Technical report	2	13	(I, J)
Indigenous Nation			
First Nations	12	75	(A, B, F–I, K–P)
Inuit	3	19	(C, D, E)
Other (Metis, Mohawk, Cree, Ojibway, etc.)	1	6	(J)
Drinking water terminology			
Drinking water	12	75	(A–G, I, K, M, O, P)
Safe drinking water	2	13	(H, L)
Both	2	13	(J, N)
Definition of health			
Reported in article	2	13	(J, M)
Cited elsewhere	3	19	(F, I, P)
Not reported	11	69	(A–E, G, H, K, L, N, O)

a Refer to Table III for key to study titles and authors.

Twelve papers described participating communities as including First Nations (12/16; A, B, F–I and K–P), three described drinking water-related health issues among Inuit communities (C–E) and one government document reviewed the effects of drinking water on health as perceived by 11 First Nation, Inuit and Métis grandmothers from several different Canadian provinces (O) (Table III).

Terminology used to describe drinking water quality in Indigenous communities was fairly consistent among the identified articles (Table IV). Two papers used both terms “drinking water” and “safe drinking water” interchangeably; two used “safe drinking water,” while most (11/16) of reviewed articles used the term “drinking water” alone. A definition of health was suggested in two articles and focused on either *a well-managed relationship with water and the* “life force” (J) or *eating uncontaminated foods and maintaining emotional well-being of families and communities* (M). In the remaining articles, health was not defined (11/16) or a citation was provided to another source (3/16).

#### Reported methods

The methodological characteristics of reviewed articles are summarized in Table V. Eight studies used a quantitative approach, while five were qualitative, and three used mixed methods (Table V). A variety of study designs were employed including community-based participatory research methodologies (5/16) case studies (4/16), cross-sectional studies (3/16), cohort studies (3/12) and theoretical research (1/16).

**Table V:** Methodological characteristics of publications included in the scoping review (n=16)

Methodological characteristic	Number (n=16)	%	Article ID numbers^a^
Research design			
Participatory research	5	31	(B, D, G, J, M)
Case study	4	25	(C, F, K, L)
Cross-sectional studies	3	19	(E, I, N)
Cohort	3	19	(A, O, P)
Theoretical research only	1	6	(H)
Research data			
Primary data	11	81	(A–C, G, I–P)
Secondary data	4	25	(E–G, O)
Not reported	2	13	(D, H)
Study type			
Quantitative	8	50	(A, B, E, G, I, L, N, O)
Qualitative	5	31	(D, F, H, J, M)
Mixed	3	19	(C, K, P)
Drinking water quality assessment			
Assessed qualitatively	5	31	(B, C, H–J)
Both qualitatively and quantitatively	7	44	(A, F, G, K, L, N, P)
Not assessed	4	25	(D, E, M, O)
Limitations to safe drinking water mentioned?			
Yes	14	87	(A–D, F–L, N–P)
No	2	13	(E, M)

a Refer to Table III for key to study titles and authors.

In the following section, we describe four methodologically diverse studies.

One community-based participatory research project gathered information on the nature, availability, utilization, perceptions and attitudes of community drinking water sources in both three Ontario and one Saskatchewan First Nation communities using an in-person household survey tool (B). Results indicated that risk perceptions differ by province on water source, health concern for tap water consumption, likelihood of reporting illness from tap water and spending more money on bottled water.

A cross-sectional study was conducted to assess perceptions of water quality, safety and changes over time as well as incidence and frequency of DWA in two First Nation communities (I). The study involved the collection of household surveys and interviews from over 900 residents of First Nations communities and 706 residents of other small communities off reserve with the aim of exploring how these communities felt about the safety of their water since the implementation of the First Nations Water and Wastewater Action Plan. Results indicated that residents of First Nations reserves are less confident about their water source, household water supply and overall water safety than non-reserve populations.

A retrospective cohort study on First Nations reserves in British Columbia (O) assessed the prevalence of hepatitis A among residents in these areas and its association with drinking water. Results indicated that increased hepatitis A incidence was associated with greater numbers of persons per housing unit. Further inspection as to the relationship with water and wastewater services was not suggested as data were insufficient to infer relationships.

A case-study example in our sample included a look at multiple dimensions of water insecurity, impacts on health and how they relate to policy changes in Inuit communities in Black Tickle, Labrador (C). Open-ended interviews with community leaders and elders, as well as focus groups with community members explored water use patterns, water quality, community health and coping strategies. The results included a local perspective defining the attributes of current health, social and political water-related issues in the communities. All major water sources were also tested for microbiological, chemical and physical contaminants and found concern with levels of disinfectant by-products and turbidity.

#### Reported data collection tools

The articles in the sample reported using questionnaires and survey instruments (A, B, E, I, N, O and P); literature reviews and case reports (C, F and H); community workshops/document analysis (K); focus groups and open-ended interviews (D, J and M); and on-site water testing (L) as primary tools (Table III). Primary data were gathered in most articles (11/16) (Table V). The number of communities that were part of these studies ranged from 1 (C, K and L) to 56 (N); however, research for the study with the most communities was focused on the water systems at each location. The largest community level study with primary data included information from 750 people in six communities (A).

Secondary data were used in four articles (including one that also collected primary data) including counts of gastrointestinal-related clinic visits obtained from health clinic records, weather and water quality results (E, F, G, O) (Table V). Two articles did not include primary data; rather, they included programme evaluations and source water protection planning outcomes with no reported primary data (D and H).

### Methodological strengths and limitations of the sample

Quantitative and mixed methods articles were examined for response rates where applicable, and risks for biases (Table III). For example, in a cohort study on the incidence of gastroenteritis in three Indigenous communities in British Columbia and the source water characteristics (O), biases and limitations were reported as selection bias, blinding and mixed water sources. Selection bias was minimized by random sampling of community clusters. Failure to blind study participants and lack of pre-screening of participants may have skewed the results of the incidences of illness. Unreported use of mixed water sources (bottled, tap and raw) made it difficult to make conclusions about source water and gastrointestinal illness, and underreporting of symptoms and the accuracy of reporting were other limitations to the study (O).

Selection bias was prominent among the studies; 4/8 quantitative studies reported selection biases due to language barriers (I), inadequate sampling and missing data (F, I, N and P), and the use of convenience sampling (I). In the mixed methods studies, 2/3 reported problems including participant's misinterpreting concepts such as risk (E) and having inadequate time to complete study tools (P). Further quantitative and mixed study concerns included poor record keeping on population statistics (B), and non-blinding, demographic and pre-screening errors for study subjects made it difficult to draw conclusions (O and P). Of the other quantitative and mixed methods studies (5/11), the lack of reporting of limitations demonstrates inadequate reflection and questionable strength of conclusions.

Considered as a whole, the sample undertook at least one investigation in each of seven provinces and three territories, missing three Atlantic Provinces. Ten articles reported that a barrier to establishing the quality of water on reserve was the access to sources for testing (A, C, F, G, I, L, M, N, O and P). Due to inconsistent delivery of water (i.e. funding shortages, and weather in remote locations meant that in some instances trucks could not delivery water), the unreported use of varied water sources meant that thorough sampling of water sources for quality testing and linking to health outcomes was not possible (8/12 reporting water quality assessment). Over 3,200 individuals were surveyed across 84 First Nation communities and 68 water systems were tested, in comparison with an estimated total of 3,100 Indigenous reserves in Canada (34).

During the analyses, we inferred further researcher limitations including access, longitudinal effects, cultural biases and language biases which were not reported in the quantitative and mixed methods studies. The lack of available baseline and prior data, longitudinal research, reliable data on source water quality, and incidence of illness or disease meant that no trends or relationships in our sample of articles could be established on water provision and quality, and health outcomes on First Nations reserves in Canada from the reported samples. At best, the quantitative sample gave unsystematic evidence for challenges in discovering relationships among water and health in First Nations reserves in Canada.

Among the qualitative studies, three concerns were reported. The first related to conditions resulting in poor drinking water quality. Uncertain water provision and poor economic conditions, which exacerbate management and infrastructural challenges in communities, were reported as a major limitation to qualitative research (i.e. the interview results would change if conducted at a different point in time because of the uncertain water situation) (D, J and M). Secondly, researchers also indicated that communication and related socio-political dimensions among the Chief and Council and local people affected the results because of the inherent mistrust of “outsiders” gathering data on reserves (D). Thirdly, there was also scrutiny towards reductionist examinations of drinking water quality, which needed dispelling before interviews and focus groups were started (D and M).

Further limitations are evident. Small sample sizes and difficulty finding acceptable definitions for key terms (i.e. gastrointestinal illness, safe drinking water and health) were reported in studies using interviews and focus groups (D and J). A noted limitation is the application of frameworks (capacity measurement, programme evaluations and source water protection practices) developed for non-Indigenous communities in First Nations reserves (D). We could only ascertain that at least 11 grandmothers from First Nations across Canada (J) and an unreported number of elders, youth and local harvesters from Ontario (M) were interviewed or participated in focus groups among the sample. Only one article reported using a decolonizing approach (M).

### Thematic analysis and study findings

The findings of reviewed articles were grouped under the following key themes: (a) drinking water quality, (b) health outcomes and (c) challenges to accessing safe drinking water. Results for each are summarized below.

#### Drinking water quality

Drinking water quality was evaluated qualitatively (i.e. asking perceptions) in seven articles (five purely qualitative articles, plus two mixed methods articles; Tables III and V). Quantitative measures of water quality (tested against established standards), including turbidity, biological (total coliform, *E. coli* most commonly), physical or other chemical contaminations (free chlorine residuals), were assessed in seven articles, while mixed methods studies frequently reported both measured water quality indicators and community perceptions. For example, results in a perceived under-funded potable water-dispensing unit (PDWU) in an Inuit community in Newfoundland and Labrador indicated quantitatively high levels of *E. coli* and qualitatively a low perception of water safety and trust of the PDWUs (C). Residents in the community had a strong distrust in the PDWU system due to animal activities around the PDWU sources. Two articles reported on specific pathogens that included bacteria (*Escherichia coli, Campylobacter jejuni, Shigella* spp., *Helicobacter pylori* and *Giardia lamblia*), viruses (*Hepatitis A*) and protozoa (*Cryptosporidium*) (L and O).

Qualitative measures of water quality were assessed in terms of risk perception in five other articles (Table III). Mixed results were found among First Nation community members when surveyed about their perceptions concerning health risks from tap water and bottled water (B, E and I). Risk perceptions for First Nations people were cautious in general, but differed by province, water source, health concerns for tap water consumption, likelihood of reporting illness from tap water, and money spent on bottled water (B, E, I, M and N). Residents of First Nations reserves were less confident about their water source, household water supply and overall water safety than non-reserve populations (B, E, I and N). Although some authors reported that participants disliked and did not trust the taste of chlorinated water (E and N), another reported that the addition of chemical treatment to water made community members feel safer (I).

#### Health outcomes associated with poor drinking water in Indigenous communities

A variety of concerns were reported about the health impacts or poor drinking water quality and are summarized in Table VI. All of the articles concluded with statements linking increased risk of negative health outcomes with poor drinking water quality. The most commonly stated health issues reported in relation to drinking water were gastrointestinal infections (12/16). The two next most commonly reported health issues (5/16) were skin problems (eczema and skin cancers) and birth defects. Other reported health problems included obesity, diabetes, hypertension, mental stress (anxiety and depression), heart diseases, liver diseases, kidney problems, neurological problems, immunopathology, cancers, thyroid conditions and infant mortality.

**Table VI:** Health outcomes related to drinking water described in publications included in the scoping review (n = 16)

Health issues described in identified articles	% (Frequency)	Article ID numbers^a^
Gastrointestinal infections	75 (12/16)	(A–C, E–H, K, L, N–P)
Birth defects and developmental problems (genetics)	31 (5/16)	(A, C, F, O, P)
Skin problems	31 (5/16)	(C, F, I, J, P)
Obesity	19 (3/16)	(C, F, P)
Diabetes	19 (3/16)	(C, F, P)
Cancers	19 (3/16)	(C, F, P)
Infant mortality	13 (2/16)	(F, P)
Mental stress	13 (2/16)	(F, P)
Neurological problems	13 (2/16)	(A, F)
Hypertension	6 (1/16)	(F)
Heart diseases	6 (1/16)	(F)
Liver diseases	6 (1/16)	(F)
Kidney problems	6 (1/16)	(F)
Immunopathy and autoimmune diseases	6 (1/16)	(F)
Thyroid disease	6 (1/16)	(A)

a Refer to Table III for key to study titles and authors.

Source water is a key concern for heath determinants. One article reported a higher risk of iodine deficiency in First Nations men in Quebec who drank spring or tap water compared with those that drank more bottled water (A), while increased skin diseases were reported in First Nations communities with over-chlorination (F). Mercury, lead, arsenic and toxic pollutants like phenols, dioxins and polycyclic aromatic hydrocarbon contamination were a reported concern (F and M). Authors noted, however, that more research is needed to determine if higher rates of infant mortality, birth defects, developmental problems, cancers and other chronic conditions could be explained by community demographics. Diet, lifestyle, environmental factors and susceptible populations (infants, the elderly, pregnant women and people with co-existing health conditions) have been implicated in the literature (35–37).

Diabetes was a significant health concern among First Nations and Inuit people identified within the scoping review (C, F and P) and other work on diabetes prevalence (38). An increase of reliance on carbonated, sugary drinks (soda pop) sold relatively cheaper than bottled water in many communities was put forward as an explanation. Although the scoping review articles did not place much water-related emphasis on the prevalence of diabetes in First Nations, Métis and Inuit populations, they have a rate of diabetes 3–5 times higher than other Canadians (39).

In summary, some health outcomes have been connected to poor drinking water services in Indigenous populations; however, due to the limitations of the research, there was not sufficient information to evaluate the potential for causal relationships between the water quality and the reported health concerns.

#### Challenges to accessing safe drinking water

Most articles (14/16) described some limitations to accessing safe drinking water within Indigenous communities (Table V). A major limitation to safe drinking water in the sample was the remote location of reserves and traditional lands. Study participants reported using raw water (occasionally, to always) from local sources (springs, lakes, wells and opportunistic “bush” waters) (A, C, D, E, F, G, I, J, L, M, O and P). Others relied on trucked-in water which was uncertain (i.e. in poor weather, or without adequate funding and personnel, water was undelivered) (C, I and K). Other problems in accessing safe water in remote locations were the training and retaining of certified water operators (C, D, K, N and P). Training programmes were offered in distant urban areas with different governing systems (D). One study reported the difficulty in retaining personnel once trained, and in retaining personnel to complete training both because of the lack of nearby support personnel, such operators were on-call every day, and because of the fear of making mistakes while operating complex water systems (K).

The differences in cultural beliefs were also noted as a major challenge to safe drinking water (E, F, J, K, L, M and O). A participant is quoted in one article stating that Indigenous teachings indicated that water provides for both the hydration of the body and giving “spirit” in each drink. The participant pondered that “anything wrapped in plastic dies… Are we feeding our people dead water?” when asked about the community's use of bottled water (J, p. 20). The misunderstanding of the values of water and how Indigenous people relate to it meant that culturally inappropriate water programmes and communication barriers prevented consistent access to safe drinking water in the perspective of community members (K, L and M). One study shed light on how culturally engaging water projects increased knowledge and development of local adaptation strategies to support better health outcomes in Indigenous communities (D). Understanding cultural knowledge of water was, therefore, a challenge to accessing safe drinking water.

Finally, formal procedures for applying for government support for improved drinking water and research on water systems were a challenge reported in the sample (B, C, D, E, F, K and N). Communities felt constrained because of their dependence on the federal government for funding for water services (B, C, F, K and N). Funding cycles for both water service applications and research grants needed to be lengthened to allow communities to gain capacity and to allow researchers to complete ethics approval processes which are lengthy and conduct meaningful community-engaged projects which take time (C, D, E and K).

## Discussion

This scoping review utilized a systematic approach to explore the nature and extent of information on health issues associated with poor drinking water in Indigenous communities in Canada. The review found 16 relevant articles following a scope of an initial pool of 518 articles. The most striking observations in this review were the paucity of literature on the topic of water and health in Indigenous communities in Canada as well as the variation in the methodologies used to assess drinking water quality and perceptions of water and health in these communities. Only one article in the sample used a decolonizing approach. Given the recommendations of the Truth and Reconciliation Commission, governments, researchers and Indigenous communities are in need of new approaches and improved relationships to move forward on issues of health and safe drinking water. Nevertheless, the findings validated previous reports describing inequalities related to the quality of drinking water and associated key health outcomes in Indigenous communities in Canada.

Contamination of water by microbial pathogens was the most commonly discussed direct risk to health and verifies research on drinking water quality and health outcomes in other contexts (38,40). There is no national surveillance system for the systematic collection of waterborne disease outbreak data (41), but recent studies described 288 recorded outbreaks of infectious disease related to drinking water in Canada over a 27-year period until 2001 (40,42). The scoping review articles revealed that the overall number of gastrointestinal infections in Indigenous communities was 26 times greater than the rest of Canada, and cases are more likely to go underreported due to different perceptions of risk and health (7–9). High incidences of other health outcomes are linked through a variety of processes to access to high-quality water (i.e. Hepatitis A and diabetes). Of particular concern were toxic pollutants in the water and their effects on children and the elderly. Lack of source water protection, governance role confusion, remote locations and unpredictable weather changes, malfunctioning water distribution systems, human error, cultural considerations and poor funding were put forth as root causes in our sample. Research on synergistic effects among anthropogenic pollutants, source water characteristics and existing diseases in populations of Indigenous people in Canada, however, is sparse.

Differences in the conceptualization of health, safe water and risk among researchers and participants were brought forward in the articles. The two articles that provided explanation of health from the participants’ point of view described health as a well-managed relationship with water (the life-force); and about eating uncontaminated foods, and emotional well-being. These concepts are very different from public agency definitions (i.e. health as the absence of chronic or infectious diseases, and injuries) (43) and are worth investigating so that researchers and participants alike are working towards the same thing. Similar misalignment of researcher and Indigenous definitions of education, housing and research designs has recently been put forward in the literature (44–47). Given the logistical challenges associated with conducting health and drinking water research with Indigenous communities, we expect that the growing body of research in this field will continue to use similar colonizing approaches, scientific definitions and non-Indigenous community standards against which quality of water and health outcomes are monitored. Enhancing Nation-to-researcher communications about the use of decolonizing research approaches is one way forward to improving the salience and legitimacy of drinking water and health research in Indigenous communities in Canada, and translating findings into public health policies that work for Indigenous people.

There are significant gaps in the knowledge of health outcomes related to drinking water in Indigenous communities. There are no longitudinal, systematic studies of drinking water and Indigenous communities across Canada. No fully agreed on indicators of drinking water safety have been catalogued or evaluated on a systematic basis, and for which researchers could create a database or link to health outcome data. Confusion exists on reserves as to whether illnesses such as gastrointestinal illnesses are related to drinking water, and there is a problem with underreporting potential drinking water-related illnesses. More could be done to educate reserve populations on potential waterborne illnesses and steps to reporting them, and to ensure health care and drinking water records are being maintained. There are no studies that focus on drinking water and health of children in Indigenous communities. Given the recognition that many adult health problems originate in childhood, these studies are acutely necessary.

To move forward on ameliorating the conditions of drinking water and health outcomes in Indigenous populations in Canada, we suggest the following recommendations that emerged from the scoping review:

- Build a coordinated network of researchers, communities, representative organizations and government agencies to conduct large cross-sectional and longitudinal studies examining the relationships between drinking water and health outcomes in Indigenous communities in Canada.
- Develop a database and management system for collating information on health outcomes related to drinking water in Indigenous populations. This can be co-created (see e.g. 48–50) to include indicators and data sets derived from multiple knowledge systems and must do so in an ethical and respectful way. Clear definitions of concepts (i.e. safe drinking water, health and risk) from Indigenous worldviews should be developed as a part of this process.
- Encourage funding agencies to put together a special call for interdisciplinary work on safe drinking water quality and quantity and health outcomes in Indigenous contexts across a variety of platforms to encourage immediate and longer-term projects targeting needs as discovered in this scoping review (i.e. widespread water quality data and content analysis of health records for “suspected” water-related illnesses on reserves as well as examining source water protection issues, community perceptions of risk and health; and policy mapping).
- Create funding opportunities to develop capacity within Indigenous communities to monitor and report drinking water safety and health outcomes and to implement strategies for ameliorating barriers and challenges to safe drinking water access.

## Conclusions

This scoping review is indicative that there is a critical need for academics to work together with Indigenous communities to understand conditions on reserve that impact drinking water quality and health outcomes and to identify solutions. Barriers and challenges exist for the communities, but also for researchers attempting to better understand the inequality. Overall, the number of studies was very small; however, the studies reported reflected a broad range of research designs and data types.

Unsatisfactory drinking water systems are common in Indigenous communities. While some research is emerging, much of the information reported to date relies heavily on data that are subject to bias as well as a number of other important reporting limitations. Future research efforts should focus on improving communications and cultural understanding, as well as increasing the numbers of communities and participants per community. Greater sample sizes are necessary to better understand the heterogeneity in experiences both within and among communities. There is a need for community-based participatory research that also applies best practices for collecting and analysing observational data when the objective is to evaluate causal associations, such as the questions raised about the impact of water quality on chronic disease in some of the studies included in this review. A step forward to improving conditions of safe drinking water would be to recognize that research must not just be credible, but also action oriented.

## References

[1] Boyd DR (2006). The water we drink: an international comparison of drinking water quality standards and guidelines. David Suzuki Foundation.

[2] Basdeo M, Bharadwaj L (2013). Beyond physical: social dimensions of the water crisis on Canada's First Nations and considerations for governance. Intl Pol J.

[3] Morrison A, Bradford L, Bharadwaj L (2015). Quantifiable progress of the First Nations Water Management Strategy, 2001–2013: ready for regulation?. Can Water Resour J.

[4] Anderson K (2010). Aboriginal women, water and health: reflections from eleven First Nations, Inuit, and Métis Grandmothers.

[5] Mascarenhas M (2007). Where the waters divide: First Nations, tainted water and environmental justice in Canada. Local Environ.

[6] Simpson L, DaSilva J, Riffel B, Sellers P (2009). The responsibilities of women. J Aborig Health.

[7] Eggertson L (2008). Despite federal promises, First Nations’ water problems persist. Can Med Assoc J.

[8] Harbinson M (2012). An analysis of water quality and human health issues in First Nations communities in Canada. ENSC 501 – Independent Environmental Study Project.

[9] Patrick RJ (2011). Uneven access to safe drinking water for First Nations in Canada: connecting health and place through source water protection. Health Place.

[10] National Collaborating Centre for Aboriginal Health (2012). The state of knowledge of Aboriginal health: a review of Aboriginal public health in Canada.

[11] Government of Canada, Health Canada (2004). Drinking water and wastewater – First Nations and Inuit Health Canada. http://www.hc-sc.gc.ca/fniah-spnia/promotion/public-publique/water-eau-eng.php.

[12] Cook C, Bakker K (2012). Water security: debating an emerging paradigm. Global Environ Change.

[13] Thomas TK, Ritter T, Bruden D, Bruce M, Byrd K, Goldberger R (2016). Impact of providing in-home water service on the rates of infectious diseases: results from four communities in Western Alaska. J Water Health.

[14] Hennessy TW, Bressler JM (2016). Improving health in the Arctic region through safe and affordable access to household running water and sewer services: an Arctic Council initiative. Int J Circumpolar Health.

[15] Arksey H, O'Malley L (2005). Scoping studies: towards a methodological framework. Int J Soc Res Meth.

[16] Pham MT, Rajić A, Greig JD, Sargeant JM, Papadopoulos A, McEwen SA (2014). A scoping review of scoping reviews: advancing the approach and enhancing the consistency. Res Synth Methods.

[17] Stevinson C, Lawlor DA (2004). Searching multiple databases for systematic reviews: added value or diminishing returns?. Complement Ther Med.

[18] Endnote^®^ X7 (2016).

[19] Mendelay 13.8© (2016). (Mendeley Ltd).

[20] Microsoft Access^®^ (2013). (Microsoft Corporation). Used with permission from Microsoft.

[21] Moher D, Liberati A, Tetzlaff J, Altman DG, The PRISMA Group (2009). Preferred reporting items for systematic reviews and meta-analyses: the PRISMA statement. PLoS Med.

[22] Tam B, Tsuji LJ, Martin ID, Liberda EN, Ayotte P, Coté S (2015). Iodine status of Eeyou Istchee community members of northern Quebec, Canada, and potential sources. Environ Sci Process Impacts.

[23] Dupont D, Waldner C, Bharadwaj L, Plummer R, Carter B, Cave K (2014). Drinking water management: health risk perceptions and choices in First Nations and non-First Nations communities in Canada. Int J Environ Res Public Health.

[24] Hanrahan M, Sarkar A, Hudson A (2014). Water insecurity in Indigenous Canada: a case study of illness, neglect, and urgency.

[25] McClymont Peace D, Myers E (2012). Community-based participatory process-climate change and health adaptation program for Northern First Nations and Inuit in Canada. Int J Circumpolar Health.

[26] Spence N, Walters D (2012). “Is it safe?” Risk perception and drinking water in a vulnerable population. Int Indigen Policy J.

[27] Harper SL, Edge VL, Schuster-Wallace CJ, Berke O, McEwen SA (2011). Weather, water quality and infectious gastrointestinal illness in two Inuit communities in Nunatsiavut, Canada: potential implications for climate change. Ecohealth.

[28] Ekos Research Associates (2011). Perceptions of drinking water quality in First Nations communities and general population.

[29] Lebel PM, Reed MG (2010). The capacity of Montreal Lake, Saskatchewan to provide safe drinking water: applying a framework for analysis. Can Water Resour J.

[30] Bernier JL, Maheux AF, Boissinot M, Picard FJ, Bissonnette L, Martin D (2009). Onsite microbiological quality monitoring of raw source water in Cree community of Mistissini. Water Qual Res J Can.

[31] Smith DW, Guest RK, Svrcek CP, Farahbakhsh K (2006). Public health evaluation of drinking water systems for First Nations reserves in Alberta, Canada. J Environ Eng Sci.

[32] Jin A, Martin JD (2003). Hepatitis A among residents of First Nations Reserves in British Columbia, 1991–1996. Can J Public Health.

[33] Maclean I (2002). Source water characteristics and the incidence of gastroenteritis in Aboriginal communities in [master's thesis].

[34] Statistics Canada (2011). Aboriginal peoples in Canada: First Nations people, Métis and Inuit. https://www12.statcan.gc.ca/nhs-enm/2011/as-sa/99-011-x/99-011-x2011001-eng.cfm.

[35] Peterson H, Torchia M (2008). Safe drinking water for rural Canadians. Can Med Assoc J.

[36] Peterson H (2001). Rural drinking water and waterborne illness.

[37] Timoney K (2007). A study of water and sediment quality as related to public health issues, Fort Chipewyan, Alberta.

[38] Government of Canada, Public Health Agency of Canada (2013). Food-borne and water-borne infections – invisible threats – The Chief Public Health Officer's report on the State of Public Health in Canada 2013: infectious disease – the never-ending threat – Public Health Agency of Canada. http://www.phac-aspc.gc.ca/cphorsphc-respcacsp/2013/food-water_alim-eau-eng.php.

[39] Government of Canada, Health Canada (2005). Diabetes – First Nations and Inuit Health Canada. http://www.hc-sc.gc.ca/fniah-spnia/diseases-maladies/diabete/index-eng.php.

[40] Charrois JW (2010). Private drinking water supplies: challenges for public health. Can Med Assoc J.

[41] Moffatt H, Struck S (2011). Water-borne disease outbreaks in Canadian small drinking water systems, small drinking water systems project. National Collaborating Centres for Public Health.

[42] Schuster CJ, Ellis AG, Robertson WJ, Charron DF, Aramini JJ, Marshall BJ (2005). Infectious disease outbreaks related to drinking water in Canada, 1974–2001. Can J Public Health.

[43] Government of Canada (2016). Public Health Agency of Canada. About the Agency.

[44] McGregor HE (2013). Situating Nunavut education with Indigenous education in Canada. Can J Educ.

[45] Christensen J (2013). Our home, our way of life”: spiritual homelessness and the sociocultural dimensions of Indigenous homelessness in the Northwest Territories (NWT), Canada. Soc Cult Geogr.

[46] Kovach M Emerging from the margins: Indigenous methodologies. Research as resistance: revisiting critical, Indigenous, and anti-oppressive approaches.

[47] Castleden H, Morgan VS, Lamb C (2012). “I spent the first year drinking tea”: exploring Canadian university researchers’ perspectives on community-based participatory research involving Indigenous peoples. Can Geogr-Geogr Can.

[48] Tobias JK, Richmond CA, Luginaah I (2013). Community-based participatory research (CBPR) with indigenous communities: producing respectful and reciprocal research. J Empir Res Hum Res Ethics.

[49] Radu I (2015). Miyupimaatisiiun in Eeyou Istchee: healing and decolonization in Chisasibi. [doctoral dissertation].

[50] Koster R, Baccar K, Lemelin RH (2012). Moving from research ON, to research WITH and FOR Indigenous communities: a critical reflection on community-based participatory research. Can Geogr.

